# Deep Sequencing of the Rat MCAO Cortexes Reveals Crucial circRNAs Involved in Early Stroke Events and Their Regulatory Networks

**DOI:** 10.1155/2021/9942537

**Published:** 2021-11-24

**Authors:** Chengtan Wang, Yuying Yang, Mengsi Xu, Fuxiu Mao, Peng Yang, Shan Yuan, Rui Gao, Shangquan Gan

**Affiliations:** ^1^State Key Laboratory of Sheep Genetic Improvement and Healthy Production, Xinjiang Academy of Agricultural and Reclamation Sciences, Shihezi, Xinjiang 832000, China; ^2^Department of Biochemistry, College of Medicine, Shihezi University, Shihezi, Xinjiang 832002, China

## Abstract

Circular RNAs (circRNAs) are highly enriched in the central nervous system and significantly involved in a range of brain-related physiological and pathological processes. Ischemic stroke is a complex disorder caused by multiple factors; however, whether brain-derived circRNAs participate in the complex regulatory networks involved in stroke pathogenesis remains unknown. Here, we successfully constructed a cerebral ischemia-injury model of middle cerebral artery occlusion (MCAO) in male Sprague-Dawley rats. Preliminary qualitative and quantitative analyses of poststroke cortical circRNAs were performed through deep sequencing, and RT-PCR and qRT-PCR were used for validation. Of the 24,858 circRNAs expressed in the rat cerebral cortex, 294 circRNAs were differentially expressed in the ipsilateral cerebral cortex between the MCAO and sham rat groups. Cluster, GO, and KEGG analyses showed enrichments of these circRNAs and their host genes in numerous biological processes and pathways closely related to stroke. We selected 106 of the 294 circRNAs and constructed a circRNA-miRNA-mRNA interaction network comprising 577 sponge miRNAs and 696 target mRNAs. In total, 15 key potential circRNAs were predicted to be involved in the posttranscriptional regulation of a series of downstream target genes, which are widely implicated in poststroke processes, such as oxidative stress, apoptosis, inflammatory response, and nerve regeneration, through the competing endogenous RNA mechanism. Thus, circRNAs appear to be involved in multilevel actions that regulate the vast network of multiple mechanisms and events that occur after a stroke. These results provide novel insights into the complex pathophysiological mechanisms of stroke.

## 1. Introduction

Ischemic stroke and its sequelae, characterized by high mortality and disability rates, present a global medical problem and public health challenge [1]. The initiating factor for ischemic stroke is tissue ischemia, caused by cerebrovascular blockage, and the resulting ischemic cascade [2, 3]. The ischemic cascade is a complex series of events influenced by multiple factors and molecules [4, 5]. Focal cerebral hypoperfusion may trigger a sequence of cellular bioenergetic failures, inducing excitotoxicity [6], oxidative stress [7], damage to microvasculature [8], disruption of blood-brain barrier function [9], and postischemic inflammation [10], ultimately leading to apoptosis [11] and necrosis [6] of neurons, neuroglia, and endothelial cells. Extensive crosstalk occurs during cascade events [6]. The ATP deficiency after stroke leads to mitochondrial damage and further combines with reperfusion injury to trigger oxidative stress in brain tissues. Oxidative stress, in turn, aggravates existing mitochondrial damage, leading to the initiation of apoptosis through the release of apoptosis-inducing proteins in the ischemic core region [11]. Necrotic neurons release large amounts of damage-associated molecular patterns, which are recognized by pattern recognition receptors, and activate resident immune cells, such as microglia, by releasing inflammatory cytokines [12]. These inflammatory cytokines also recruit macrophages to initiate the so-called inflammatory cascade waterfall effect that further aggravates tissue damage in the brain [13–15]. Notably, ischemic cascades can either promote or inhibit each other. Reactive oxygen species (ROS) attack and oxidize phospholipids in cell membranes. These oxidized phospholipids are recognized by CD36, which, in turn, promotes the activation of Toll-like receptor 2 and the triggering of the downstream inflammatory response [16]. Thus, the elevated inflammatory response synergistically promotes ROS production and exacerbates oxidative stress injury [17–19], whereas moderate inflammatory responses or inflammatory factors, such as TNF-*α* and IL-1, induce extracellular superoxide dismutase, which can scavenge extracellular superoxide anions [20]. In these series of pathological processes, interactions between numerous biological molecules at different levels and spatio-temporal expression result in a complex cascade that includes protein-protein interactions, gene coexpression, and competing endogenous RNA (ceRNA) regulatory networks. The collective data provide a new perspective on ischemic stroke. They reveal that multiple pathways participate in the pathophysiological processes through intertwined biological cascade networks via interactions and regulatory effects at different molecular spatio-temporal levelof expressions [21, 22]. Thus, a comprehensive evaluation of the biochemical cascade networks associated with poststroke injury should advance our basic understanding of the pathophysiological processes of stroke. They will also provide new targets of treatment strategies to prevent the progression of complex diseases.

Circular (circ) RNA is a class of noncoding RNA molecules, and each member contains a covalent circular structure formed by alternative back-splicing [23]. They were originally identified in plant genomes and hepatitis viruses. These RNA molecules are stable, even in the presence of RNase R [24, 25], because they possess a circular structure and lack the 3′ end cap and poly A tail [23, 26]. Moreover, circRNAs are highly conserved among mammals [23, 27] and display tissue-specific expression [28]. Brain tissues, particularly the cortex and cerebellum, are regions highly enriched with mammalian circRNAs [29, 30]. Moreover, the conservation of central nervous circRNAs is interrelated with their high expression patterns [29]. These properties offer potential applications in the diagnosis and treatment of neurological disorders.

circRNAs have multiple biological functions. First, circRNAs harboring a large number of miRNA-binding sites [25, 31] serve as “miRNA sponges” that absorb a large number of miRNA molecules, leading to the regulated expression of their downstream target genes [25, 32, 33]. Moreover, circRNAs participate in numerous complex diseases, such as neurological disorders, cancer, and cardio-cerebrovascular diseases, through the ceRNA mechanism [34–37]. Several studies have reported the significant ceRNA-dependent involvement of circRNAs in stroke development. For instance, Han and coworkers showed that the expression of circHECTD1 is markedly altered in MCAO mice and improves stroke outcome by inhibiting the activation and autophagy of astrocytes [32]. Bai et al. demonstrated that circDLGAP4 regulates HECTD1 expression through miRNA-143, affecting the integrity of the blood-brain barrier for the further regulation of cerebral ischemia-reperfusion injury [9]. circRNAs play complex regulatory roles via the ceRNA mechanism. One circRNA can inhibit multiple miRNAs, and one miRNA cannot only modulate several downstream target genes' expression levels but can also be jointly inhibited by one or more circRNAs, thereby generating a complex ceRNA regulatory network. Second, circRNAs can also promote or repress the expression of their linear host genes. For instance, during circRNA synthesis, the nonclassical reverse splicing during circRNA synthesis competes with the classical splicing of their host genes' mRNAs, affecting the expression of the parental genes [38, 39]. Additionally circRNAs can interact with U1 small ribonucleoproteins (snRNPs) and RNA polymerase II to act on promoter regions of host genes and eventually enhance their transcription [24, 40]. Therefore, circRNAs may influence stroke pathophysiology through potentially extensive regulatory mechanisms in a diverse range of networks. However, the integrated regulatory network of circRNA participation in response to ischemic is poorly understood at present.

In this study, we induced cerebral ischemia-reperfusion injury with the MCAO method and analyzed the differential cortical circRNAs expression profiles in the cortex between the MCAO and sham groups at an early postischemia injury (24 h) stage using high-throughput sequencing. Divergent RT-PCR and qPCR were employed to validate the sequenced circRNAs with back-splicing and the differentially expressed circRNAs (DECs), respectively. GO and KEGG enrichment analyses were applied to predict the involvement of host genes of DECs participating in disease signal transduction pathways and biological processes. We further predicted the miRNAs adsorbed by DECs and their targeted mRNAs, thereby constructing a circRNA-miRNA-mRNA expression regulatory network potentially involved in the response to oxidative stress, apoptosis, angiogenesis, and inflammation after stroke. In summary, a comprehensive analysis of the role of circRNAs in stroke occurrence and development, including their host gene functions and ceRNA regulatory networks, provided novel insights useful for the prevention and treatment of stroke.

## 2. Materials and Methods

### 2.1. Animals and Groups

#### 2.1.1. Experimental Rats

Sprague-Dawley rats (8–9-week-old males weighing 240 ± 20 g) were purchased from the Xinjiang Center for Disease Control and Prevention. The 22 rats were numbered and randomly assigned to two groups using a random number generator. In total, 11 rats were included in both the MCAO group and sham operation groups. The number of rats used for the experiments was determined by the need for sequencing and validation requirements as follows: five for RNA-seq, three for TTC staining, and three for H&E staining. A laser was used to mark the tail of each rat to minimize potential confounders. Experiments were conducted following the Ethics Guidelines for Animal Experiments of the First Affiliated Hospital, School Medicine of Shihezi University (Xinjiang Province, China).

#### 2.1.2. Model

Middle cerebral artery occlusion (MCAO) in rat brain was generated in accordance with the method of Longa et al. [9, 32]. Rats were anesthetized with pentobarbital sodium (40 mg/kg) and fixed in the supine position. After routine skin sterilization and preparation, a small incision (~25 mm) was made on the left side of the midline of the neck to bluntly separate the carotid muscles and expose the internal and external carotid arteries, as well as the carotid bifurcation. A small incision was made in the external carotid artery, and a silicone-coated MCAO plug (Jialing Biotechnology Co., Ltd., Guangzhou, Guangdong, China) was carefully inserted into the middle cerebral artery via the internal carotid artery until slight resistance stopped the embolization. After 2 h of ischemia, the plug was removed for reperfusion. MCAO surgery led to infarctions of the left middle cerebral arteries in rats. To exclude the effects of the surgical procedure on the neurobehaviors of rats, we established a sham-operated group in which the surgical procedures were identical to those for the MCAO group, except that the plug did not enter the internal carotid artery.

### 2.2. Neurobehavioral Assessments

Scoring was performed by investigators blinded to the experiment at 24 h after reperfusion following MCAO using the Longa scores and elevated body swing test (EBST) scoring criteria to ensure the maximum validity and success of the model [41, 42]. The MCAO model group, having Longa score > 1, EBST score > 0.9, and significant pallor edema visible on the ischemic side of the brain at the time of brain retrieval, was used as a valid sample. To exclude the effects of cerebral hemorrhage on neurobehavior, the inclusion criteria for rats in the experimental group also included the absence of subarachnoid hemorrhaging.

### 2.3. Histological Procedures of MCAO

#### 2.3.1. 2,3,5-Triphenyltetrazolium Chloride (TTC) Staining

Immediately after the removal, the brains were frozen for 20 min at -20°C. Afterwards, whole-brain tissue was consecutively sectioned into coronal slices of 2 mm thickness. Sections were stained by immersion in 2% TTC staining solution (Sigma-Aldrich, St. Louis, MO, USA) for 30 min at 37°C and placed in 4% paraformaldehyde (Sigma-Aldrich) for 24 h to determine the size and extent of the infarcted area.

#### 2.3.2. Hematoxylin and Eosin (H&E) Staining

The thoracic cavity was opened, and the left atrial appendage was cut open and perfused with saline. When the effluent turned pink, 4% paraformaldehyde (Sigma-Aldrich, St. Louis, MO, USA) was used to initially fix rat brain tissue. Then, brain tissue was removed, placed in 4% paraformaldehyde, and fixed for 24 h at -4°C before embedding in paraffin. Next, paraffin-embedded brain tissue was cut into 5 *μ*m sections and stained using hematoxylin and eosin. Images of stained paraffin sections were obtained under a microscope (Olympus, Japan).

### 2.4. Total RNA Extraction and circRNA Sequencing

Five rats in the MCAO group and five in the sham-operated group numbered M01–5 (MCAO group) and S01–5 (sham-operated group), respectively, that met the criteria were selected. After being anesthetized with pentobarbital sodium, rats were decapitated, and the cerebral cortex was separated on ice and stored in liquid nitrogen. Total RNA was extracted using a TRIzol reagent kit (Invitrogen, Carlsbad, CA, USA), and quality control was performed using an Agilent 2100 instrument. The Ribo-Zero rRNA Removal Kit (Epicentre, Madison, WI, USA) and RNase R were used to remove ribosomal and linear RNA, respectively, from high-quality total RNA. Total RNA sequencing was performed on the Illumina platform, and raw sequenced data was processed to remove adapters and low-quality sequences. High-quality clean data were compared and annotated to the rat reference genome (ftp://ftp.ensembl.org/pub/release-95/fasta/rattus_norvegicus/) using the BWA tool [43]. To avoid false positives created by a single software prediction, both CIRI and find_circ software were used to scan sequencing data and their intersecting results were taken as confident circRNA predictions [44, 45].

### 2.5. Identification and Quantification of circRNAs

Candidate circRNAs were selected on the basis of high, medium, and low expression patterns for qualitative and quantitative validation. RT-PCR with divergent primers was used to amplify the region containing the back-splicing junction to verify the loop-forming characteristics of circRNAs. Total RNA was reverse-transcribed with random primers (Promega, Madison, WI, USA), and reverse transcriptase was from GoScript™ Enzyme Mix (Promega). Primers for amplification were synthesized by Sangon Biotech (Shanghai) Co., Ltd. (Shanghai, China). The primer sequences are shown in Table 1. EmeraldAmp MAX PCR Master Mix (RR320A, Takara, Japan) was used for PCR with the following parameters: initial denaturation at 94°C for 3 min, followed by 35 cycles of 94°C for 30 s, 60°C for 30 s, and 72°C for 30 s.

qRT-PCR primers and reverse transcription reactions were the same as those used for RT-PCR. qRT-PCR amplification was performed using a TB Green®Premix Ex Taq™ kit (RR820A, Takara, Japan). qRT-PCR conditions were as follows: 50°C for 2 min, then initial denaturation at 95°C for 10 min, followed by 40 cycles of 95°C for 15 s and 65°C for 30 s. The dissociation curve started at 60°C.

### 2.6. Screening and Validation of DECs

We standardized the sequencing data that were mapped to the genome and identified as circRNAs using specific software. Standardized data were expressed as spliced reads per billion mapping (SRPBM) values. The circRNAs differentially expressed between the two groups were subsequently screened using the DESeq2 package in R. Screening criteria were set as |FC | ≥1.5 and *P* value < 0.05. DECs were identified using qRT-PCR performed as described above.

### 2.7. GO and KEGG Enrichment Analyses

The GO annotations were performed using a cluster profile of the screened DECs. The KEGG pathway analysis of DECs used the KOBAS online analysis database (http://kobas.cbi.pku.edu.cn/). In this study, we analyzed the host genes of the DECs that were significantly up- and downregulated as determined from integrated RNA-seq data, and a *P* value of < 0.05 was considered statistically significant.

### 2.8. Construction of circRNA-miRNA-mRNA Regulatory Networks

The key differential circRNAs, adsorbed miRNAs, and target mRNAs were screened using TargetScan software with screening criteria of at least one 8mer seed matching sequence and context score percentile > 90. Deep sequencing of miRNAs and mRNAs was performed using the Illumina platform (data not shown). Expression patterns of circRNAs and adsorbed miRNAs, as well as their targeted mRNAs, were analyzed, and the miRNA and mRNA data that fit circRNA/miRNA and mRNA/miRNA complementary expression patterns were retained. Cytoscape 3.6.1 software was applied to construct circRNA-miRNA-mRNA regulatory network.

### 2.9. Statistical Analyses

All the data are presented as the means ± SEMs. Group means were compared by one-way analysis of variance followed by LSD tests using the R package and GraphPad Prism 6.01 (GraphPad, Inc., La Jolla, CA, USA). Assays were performed in triplicate, and all the experiments were repeated three times. *P* < 0.05 was considered statistically significant. All the qRT-PCR reactions were performed with three biological replicates, and expression levels were calculated relative to GAPDH expression using the 2^−ΔΔCT^ method.

## 3. Results

### 3.1. Successful Establishment and Assessment of an MCAO Model for Cerebral Injury following Ischemia and Reperfusion

**Figure 1:**
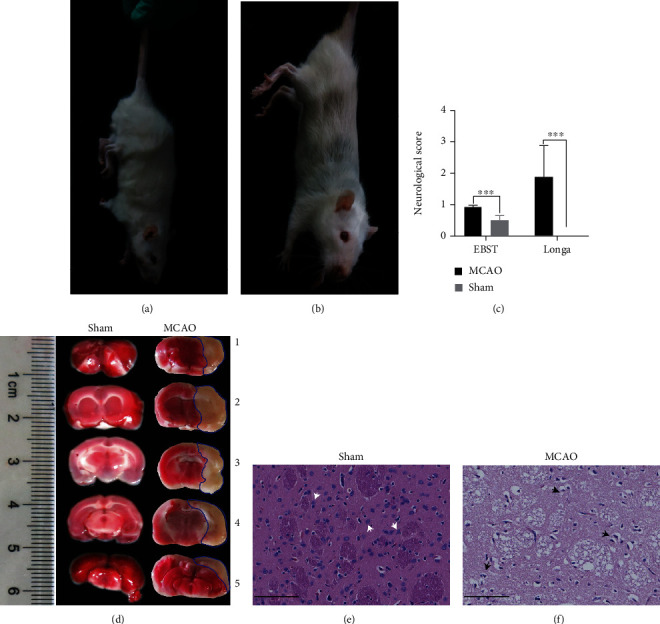
Construction and evaluation of MCAO model for brain injury following ischemia and reperfusion. (a) MCAO rat behavior observations. Longa rating of 1 confirmed the success of our MOCA rat model. Behavior manifested as mild neurological deficit symptoms, whereby forelimbs could not be fully extended. (b) Sham rat behavior observations. Forelimbs could be naturally extended without neurobehavioral deficits. (c) Longa and EBST scores. Data are expressed as the means ± SEMs and compared using Student's *t*-test (^∗∗∗^*P* < 0.001 versus sham, *n* = 5 mice per group with triplicate biological replicates). (d) TTC-stained brain sections. Ischemic area stained white (lined with blue) and the intact area stained red. Left: representative coronal brain sections of the sham group; right: MCAO group at 2 h MCAO followed by 24 h of reperfusion. (e, f) H&E-stained brain slices of sham and MCAO groups, respectively (scale bar = 100 *μ*m). Neurons in (e) were tightly aligned with moderate and distinct nucleoli. The white arrows indicate undamaged nerve cells with intact structural morphology. Owing to the presence of vacuolation and mesenchymal edema, staining of the infarcted area was lighter than that of the sham group. The black arrows indicate pathological abnormalities with loosely arranged neurons, pyknotic nuclei, nuclear lysis, and vacuole-like degeneration.

To systematically investigate the genes and signaling pathways activated in response to pathological changes of cerebral hypoxia-ischemia, we successfully constructed a rat MCAO model capable of simulating an early stroke. Neurobehaviorally, rats in the MCAO group showed an inability to straighten forelimbs after cerebral infarction, and they had no desire to grasp or land on the ground. In contrast, the sham group showed no signs of cerebral infarction. Their forelimbs were able to straighten naturally and the rats had a strong desire to grasp the ground (Figures 1(a) and 1(b)). Longa and EBST scores further indicated a more pronounced neurobehavioral deficit in the MCAO group (Figure 1(c), Table S1). Triphenyl tetrazolium chloride (TTC) staining was performed on the whole brains, and obvious morphological changes were observed in the brain tissues from the MCAO group compared with the sham group. The cerebral infarction areas after MCAO were edematous and pale in color, whereas brain sections of the corresponding region in the sham group were ruddy in color without obvious infarcted lesions (Figure 1(d)). After stroke, the cortex was the most sensitive tissue and was significantly affected by hypoxia-ischemia. Therefore, we performed H&E staining on the cerebral cortexes of postoperative rats. The infarcted areas in MCAO rats showed pathological changes, including structural laxity, vacuolar-like degeneration accompanied by nuclear solidification, and decreased numbers of neurons (Figures 1(e) and 1(f)). The neurobehavioral and morphological test results clearly indicated that rats in the MCAO group had typical characteristics and manifestations of cerebral ischemia and reperfusion injury. Thus, the experimental model successfully simulated cerebral ischemia and reperfusion injury, supporting its utility in the subsequent experiments.

### 3.2. Identification and Characterization of circRNAs in the Cortexes of the MCAO and Sham Groups

To determine the numbers and types of circRNAs expressed in the rat cortex and to identify those responding to the pathological changes of cerebral hypoxia-ischemia, we performed deep sequencing of circRNAs from 10 cortex samples of MCAO and sham rats. In total, 420,636,508 and 520,236,256 raw reads were generated in the cerebral cortexes of the MCAO and sham groups, respectively. More than 99.45% and 99.65% of the clean reads could be mapped to the rat genome after removing adapters and low-quality sequences (Table S2). To improve the accuracy of circRNA identification, CIRI and find_circ software were used to predict circRNAs, and circRNAs identified by both were selected. A total of 24,858 circRNAs were identified, among which only 18.74% were known circRNAs and the information of known circRNAs (Table S3). To facilitate future studies on circRNAs in the cerebral cortex, we unified and renamed the nomenclature and annotations of known and novel circRNAs cloned in this study (Table S4). The distribution of the circRNA reads on different chromosomes was determined (Figure S1A). The circRNA distribution on chromosomes was correlated with chromosomal gene density, with the Y chromosome having the lowest gene density and lowest proportion of distributed circRNA reads (Figure S1A). In terms of length, circRNAs < 3,000 nt were most frequently located in exonic regions, whereas circRNAs > 3,000 nt mostly originated from the intergenic and intron regions (Figure S1B).

**Figure 2:**
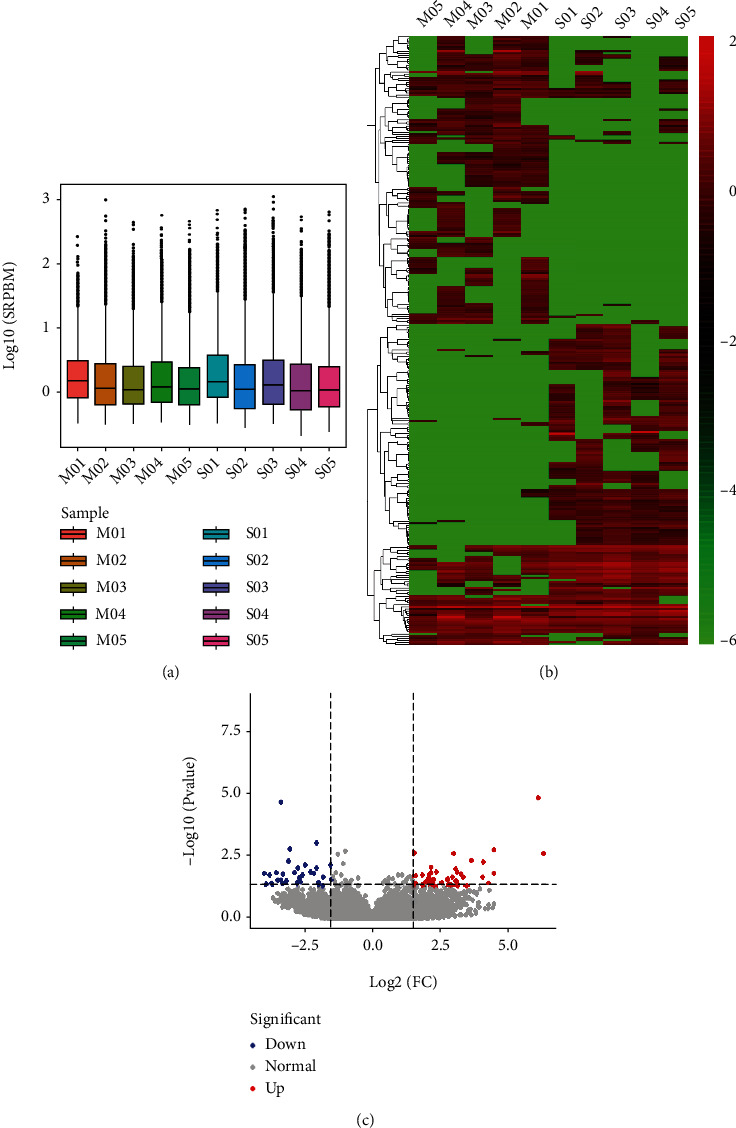
Expression analysis of circRNAs between the MCAO and sham groups. (a) Box plot describing expression abundance of circRNAs in each sample. The *x*-axis represents the sample, and *y*-axis the logarithm value of normalized sample expression with the spliced reads per billion mapping algorithm. (b) Volcano plot exhibiting DECs between the two groups. Blue and red dots represent downregulated and upregulated DECs in MCAO compared to the sham group (*P* < 0.05), respectively. Gray dots represent circRNAs with equal expression level between the two groups. (c) Clustering heat map illustrating distinguishable expression profiles of circRNAs between the two groups. Red and green colors represent fold changes of upregulated and downregulated circRNAs, respectively, whereas black signifies circRNAs with unchanged expression levels. The colors from green to red indicate increased relative circRNA expression. M01–5: cortex samples in the MCAO group; S01–5: cortex samples in the sham group.

To screen for reliable DECs, the sequencing data of each sample were examined for consistency and dispersion. Box plots showed that the expression levels of the samples were relatively concentrated, with no obvious outliers, indicating that the sequencing data were reproducible and could be used for the subsequent analysis of DECs (Figure 2(a)). Screening experiments revealed that only 1% of the circRNAs were significantly differentially expressed between the two groups. As depicted in a volcano plot (Figure 2(b)), 99% of the circRNAs displayed nonsignificant differential expression. A further cluster analysis of 294 DECs showed that the differences in the expression characteristics between the two groups were significant, and DECs in the same group had similar expression characteristics (Figure 2(c)). Compared with the sham-operated group, there were 158 up- and 136 downregulated circRNAs in the rat cortex after MCAO (Table S5). Of which, 106 belonged to the circRNAs coexpressed between the two groups.

**Figure 3:**
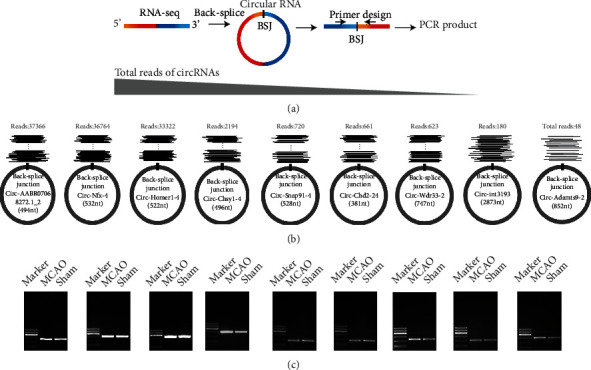
RT-PCR validation of selected circRNAs. (a) Schematic diagram of the circRNA validation strategy with divergent primers. (b) Nine novel representative circRNAs with decreasing supporting read counts. The long gray triangle represents the read expression level of circRNAs, and the supporting RNA-seq data of every read includes the back-splice site. (c) Identification of selected circRNAs through agarose gel electrophoresis of divergent amplification products. From upper to lower, the 100 bp DNA ladder includes six fragments: 100, 200, 300, 400, 500, and 600 bp. MCAO: cDNA templates derived from the MCAO group; Sham: cDNA templates derived from the sham control group.

**Figure 4:**
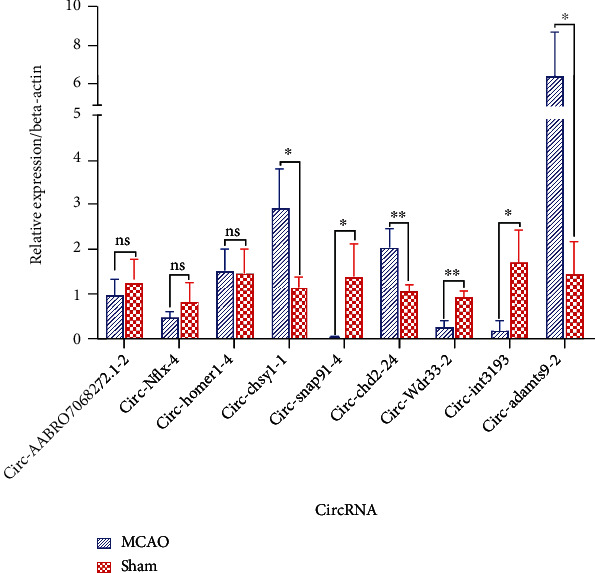
Quantitative real-time PCR (qPCR) validation of nine randomly selected circular RNAs. Nine circRNAs with nonsignificantly or significantly different expression patterns between the two groups as assessed by RNA-seq were selected. Expression patterns of circRNAs were verified using qPCR, which were consistent with the sequencing data. Data are presented as the means ± SEMs (*n* = 3). ^∗^*P* < 0.05 and ^∗∗^*p* < 0.01; two-tailed paired *t*-test.

To validate the accuracy of the circRNA sequencing data, we classified the sequenced circRNAs into high-, medium-, and low-expression groups. Three circRNAs from each group were randomly selected for RT-PCR and qRT-PCR analyses. To confirm the typically circular characteristics of nine selected circRNAs, divergent primers were specifically designed to amplify the circRNA region spanning the back-splice junction sequence (Figures 3(a) and 3(b)), and PCR products of the expected size were successfully amplified using divergent primers for each circRNA (Figure 3(c)). A further quantitative analysis of the selected circRNAs revealed consistent expression trends in qRT-PCR and sequencing reads (Figure 4), indicative of reliable sequencing data.

### 3.3. Host Gene GO and KEGG Pathway Enrichment Analyses of DECs

**Figure 5:**
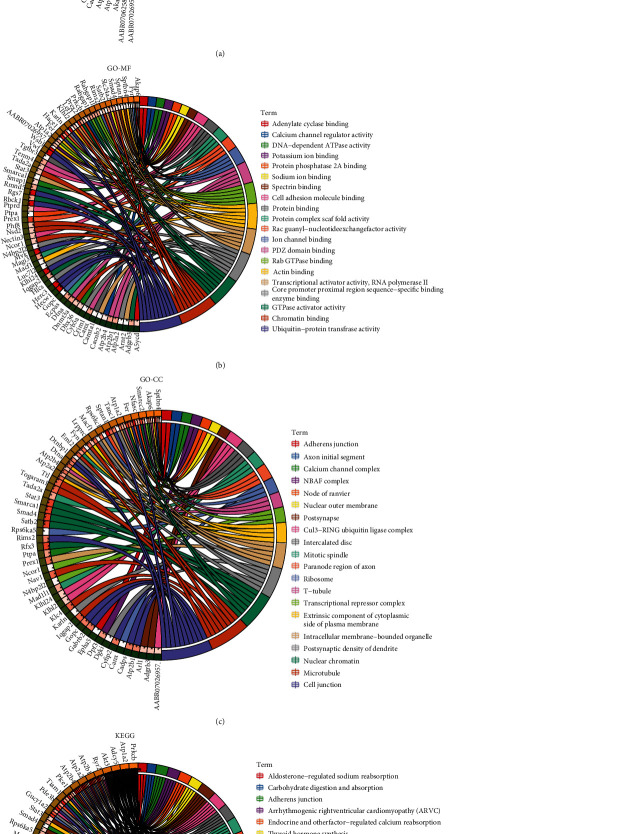
GO and KEGG analyses of host genes of DECs. (a–d) Circos plot diagrams showing the top 20 enriched (a) biological process GO terms, (b) molecular function GO terms, (c) cellular component GO terms, and (d) KEGG pathways.

Because circRNAs are closely related to the expression and function of their host genes [46], analyzing the function of the host gene is necessary to determine the specific role and regulatory network of the complementary circRNA. A GO analysis of mRNAs transcribed by the host genes of DECs revealed gene enrichment for each of the top 20 terms in the GO branches of biological process (BP) (Figure 5(a) and Table S6), molecular function (MF) (Figure 5(b) and Table S7), and cellular component (CC) (Figure 5(c) and Table S8). All the highly enriched GO terms in this study were strongly associated with changes in cerebral ischemic and hypoxic stress, brain and nerve development, and neurological disorders. Among these, the terms “calcium ion transmembrane transport of the BP branch,” “calcium channel regulator activity of the MF branch,” and “calcium channel complex of the CC branch” were related to calcium ion transmembrane transport. The disruption of calcium transport across membranes was directly associated with pathophysiological processes including the ischemic cascade of mitochondrial injury, oxidative stress, and inflammatory responses after stroke. Moreover, the terms “protein ubiquitination” in the BP branch, “ubiquitin-protein transferase activity” in the MF branch, and “Cul3-RING ubiquitin ligase complex” in the CC branch are associated with protein ubiquitination, considered a key process in the ischemic cascade response after stroke, that has strong connections to pathophysiological processes such as apoptosis, autophagy, and inflammation. The KEGG analysis further suggested that these host genes had an impact on the physiological activities of nerves. The highly enriched top 20 key KEGG pathways included adrenergic signaling in cardiomyocytes, the cAMP signaling pathway, and the cGMP-PKG signaling pathway (Figure 5(d) and Table S9). Among these pathways, cAMP, cGMP-PKG, PI3K-Akt, and MAPK signals are important in stroke development and progression. These results strongly suggested that circRNAs are highly involved in the pathogenesis of stroke.

### 3.4. Construction of the circRNA-miRNA-mRNA Regulatory Interaction Network

To further narrow down the scope of candidate DECs and construct a biologically meaningful circRNA regulatory network, we removed circRNAs having a low expression or low frequency. In total, 106 DECs coexpressed in the two groups were used to construct a ceRNA regulatory network. We used TargetScan software to select the canonical site type (8 mer) and context scores > 90 to predict miRNAs adsorbed by circRNAs as well as mRNAs targeted by miRNAs were subjected to stringent screening criteria. Consequently, 651 adsorbed miRNAs and 10,822 target genes were identified (Tables S10 and S11).

**Figure 6:**
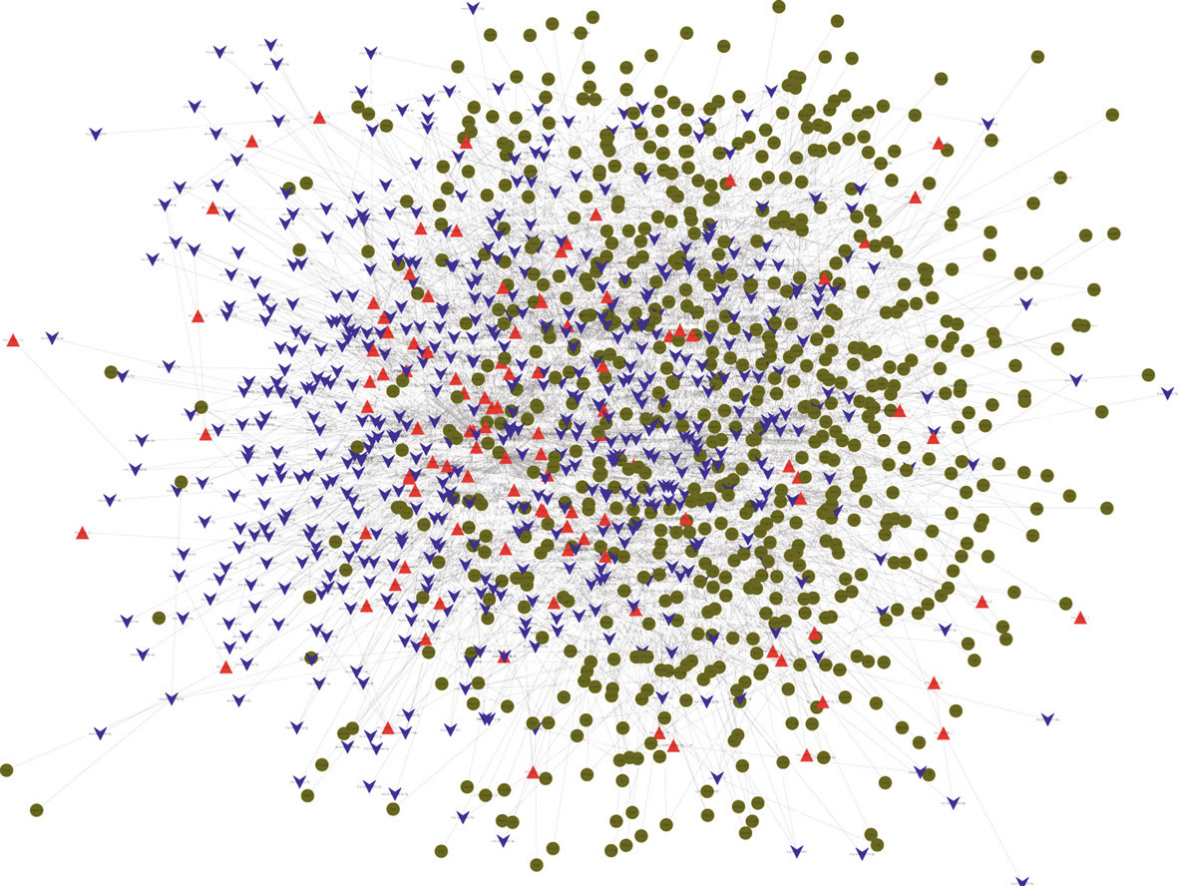
circRNA-miRNA-mRNA regulatory network analysis. Red triangles, blue arrows, and dark green circles represent DECs, harbored miRNAs, and targeted mRNAs, respectively.

To improve the confidence ratio of screened miRNAs and mRNAs, we further reduced the numbers of miRNAs adsorbed by circRNAs and of mRNAs targeted by miRNAs. With the aid of the expression profiling data on circRNAs, miRNAs, and mRNAs, we eliminated some miRNAs and mRNAs that had expression patterns that did not conform to the inverse expression relationship, leading to reductions in the numbers of candidate miRNAs and target genes to 577 and 696, respectively. A circRNA-miRNA-mRNA regulatory network harboring 106 circRNAs, 577 miRNAs, and 696 mRNAs was ultimately constructed (Figure 6, Table S10). Data from mRNA sequencing showed that >90% of the 696 mRNAs were highly expressed in the cortex, and ATPase Na^+^/K^+^ transporting subunit alpha 2, the most highly expressed gene, had 490,991 reads. There were 285 and 25 mRNAs with read numbers > 100,000 and >10,000, respectively. Only 68 mRNAs had <1,000 reads, and interleukin 17 receptor B had the lowest expression level, with 365 reads (Table S11). We additionally performed a tissue enrichment analysis using the TissueEnrich (https://tissueenrich.gdcb.iastate.edu/) program for the 696 selected genes, and they were most highly enriched in the cortex (Figure S2).

We ranked circRNAs on the basis of the number of adsorbed miRNAs and miRNA-regulated genes. The top 10 circRNAs having the most abundant gene information were circ_Wdr17_10, circ_Kalrn_2, circ_Bicd1_2, circ_intergenic_3243, circ_Atp6ap2_4, circ_Ankle2_1, circ_Bcat1_2, circ_Camta1_9, circ_Tmprss4_1, and circ_Tbcd_12. As an example, circ_Wdr17_10 adsorbed up to 216 miRNAs and 454 target mRNAs. These key circRNAs were associated with a large range of key functional genes involved in ischemic injury, such as *HIF-1*, *Bcl-2*, and *MMP-9*, in response to stroke through the adsorption of miRNAs and the regulation of target genes. Our regulatory network thus validated the involvement of circRNAs in stroke through the direct regulation of adsorbed miRNAs and targeted mRNAs from a comprehensive and multifaceted perspective.

### 3.5. Biological Pathways of 15 Key circRNAs and Their Regulatory Roles in the MCAO-Induced Stroke

**Figure 7:**
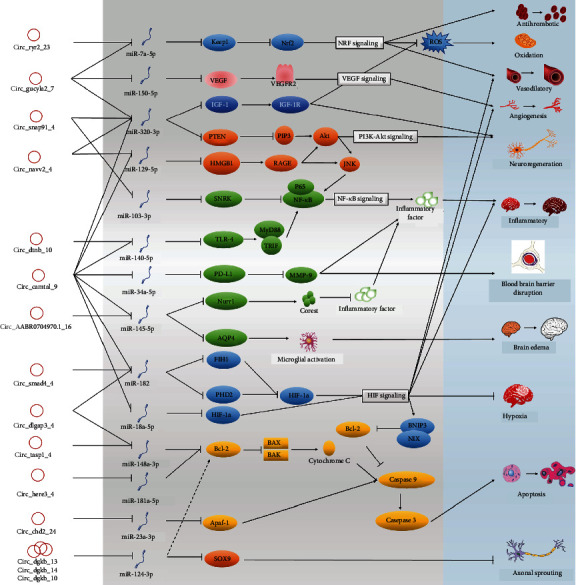
Key candidate circRNAs and their putative biological pathways involved in ischemia and reperfusion injury. The white vertical column lists the key DECs, the light and darker gray vertical columns show the harbored miRNAs and mRNA targets that play important roles in neuroinjury, respectively, and the blue vertical column lists the corresponding biological mechanisms verified in brain injury following ischemia and reperfusion.

We further screened the key DECs involved in detailed biological pathways in the pathophysiological process after stroke using the expression regulatory network consisting of 106 circRNAs, 577 miRNAs and 696 mRNA. In total, 15 circRNAs, 14 miRNAs and 16 mRNAs, generating a precise circRNA-miRNA-mRNA regulatory network (Figure 7), were finally selected after referring to the existing literature on miRNAs and mRNAs involved in stroke. Among them, the 15 circRNAs were potentially involved in a series of biological events, such as anticoagulant response, oxidative stress, vasodilation, vascular regeneration, nerve regeneration, poststroke inflammatory response, blood-brain barrier damage, cerebral edema, cerebral hypoxia, apoptosis, and axonal growth, through their linkage with adsorbed miRNAs and miRNA-mediated mRNAs. Like the binding of miRNA with mRNA, circRNAs adsorb miRNAs in “one to multiple” and “multiple to one” patterns. For instance, circ_Camtal_9 simultaneously adsorbs miR-320-3p, miR-129-5p, miR-140-5p, miR-34a-5p, miR-145-5p, miR-182, and miR-18a-5p, whereas three circRNAs of the circ_Dgkb family (circ_Dgkb_10, circ_Dgkb_13, and circ_Dgkb_14) coadsorb miR-124-3p. Our results showed that circ_Ryr2_23, circ_Gucy1a2_7, circ_Camta1_9, circ_Smad4_4, and circ_Dlgap3_1 play pivotal roles in brain hypoxic responses and neuronal oxidative stress through access to the Hif-1, Nrf, and VEGF signaling pathways (Figure 7). circ_Snap91_4, circ_Nav2_4, circ_Dntb_10, and circ_Camta1_9 regulated the inflammatory responses after stroke through the adsorption of miR-320-3p, miR-129-5p, miR-103-3p, miR-140-5p, miR-34a-5p, and miR-345-5p, as well as their targeted social inflammatory proteins, TRL-4, PD-L1, Nurr1, Snrk, and AQP4. In addition, circ_Dlgap3_1, circ_Tasp1_7, circ_Herc3_2, and circ_Chd2_24 targeted the antiapoptotic protein, Bcl-2, as well as apoptotic protease activating factor 1, supporting their participation in the apoptotic process after stroke. Although the regulatory networks are complex and dynamic, their functions are ultimately linked to biological pathways closely related to stroke, such as oxidative stress, neurovascular regeneration, inflammation, blood-brain barrier integrity, cerebral hypoxia, and cerebral edema.

## 4. Discussion

The MCAO model is widely utilized in the study of ischemic stroke. Following the previously documented method of MCAO model preparation [32], we prepared a rat MCAO model exhibiting significant neurobehavioral deficit characteristics (Longa score > 1 and EBST score > 0.9). Only male rats were selected to use in our MCAO study after considering that estrogen has a nonnegligible effect on brain injury. In TTC staining experiments, rats in the MCAO group displayed distinct areas of pale ischemic infarction. After H&E staining, the cerebral infarction areas in model rats showed structural laxity of brain tissues, disordered cell arrangements, decreased neuron numbers, and obvious infarct-like changes (vacuolar degeneration and pyknosis). The results of the neurobehavioral scoring and histological analyses were sufficient to support successful model construction and its potential utility in the analytical validation.

Limited studies have focused on the roles of circRNAs in stroke. Duan and coworkers conducted high-throughput sequencing of whole-brain tissues on the infarcted side 4 days after MCAO in rats [47]. Li et al. [48] analyzed circRNAs in thalamic tissues on the infarcted side 7 and 14 days after focal cerebral infarction in mice, and the Mehta group used microarrays to detect changes in whole-brain circRNA expression patterns at 6, 12, and 24 h after post-MCAO reperfusion in mice [49]. Wouters and colleagues demonstrated a clear correlation between stroke severity in the early stages, especially at 24 h of onset, and stroke recovery [50], and they validated the importance of early stroke injury changes and interventions for stroke management [51]. In RNA-seq experiments, often a minimum of 3 biological replicates per group are employed. However, this sample number is not fixed and many more replicates may be needed depending on the question of interest, the variance in the system being examined, as well as the statistical power that the designers wish to achieve [47–49]. In this study, morphological methods were applied to ensure the accuracy and representativeness of employed samples, and five biological replicates per group finally were identified after quality filtering of the sequencing data (Figure 2(a)). In the present study, we applied high-throughput circRNA sequencing to detect cortical circRNA expression patterns in rats 24 h after MCAO. The most susceptible regions of the brain to cerebral ischemia are the hippocampus and cortex, and data mining for circRNAs in the cortex is therefore valuable in establishing the mechanisms underlying damage and self-repair poststroke. A large number of studies have shown that the 24-hour time point is the most severe time point for nerve damage after cerebral ischemia and reperfusion injury [50, 52–55]. We detected 24,858 circRNAs, among which 20,199 were novel and 4,659 were known. Ultimately, 294 DECs were screened. Nine of the circRNAs having different sequencing reads were randomly selected, and their back-splice junctions were validated by PCR amplification with divergent primers. Subsequent qPCR analyses confirmed that the expression trends of these nine circRNAs were consistent with high-throughput sequencing data, supporting the reliability of our circRNA sequencing results.

circRNAs can affect the expression of host genes in two ways. The back-splicing mechanism of circRNA during synthesis competes with the classical splicing mechanism of synthetic mRNAs [38, 56]. circRNAs additionally enhance host gene transcription by interacting with U1 small ribonucleoproteins and RNA polymerase II in the promoter region [24, 40]. Therefore, to clarify the specific roles of circRNAs in poststroke, we performed GO and KEGG analyses of host genes of circRNAs that had been significantly altered between the two groups. In the BP and MF branches of the GO analysis, enrichment categories included protein ubiquitination, calcium ion transmembrane transport, Rac guanyl nucleotide exchange factor activity, and protein complex scaffold activity. Abnormal transport of calcium ions across membranes is a cause of secondary injuries, such as oxidative stress damage, inflammatory responses, apoptosis, and necrosis, after stroke [57]. Alterations in the steady state of calcium ions have direct impacts on the formation and recovery of the penumbra, and the existence of a penumbra indicates that therapeutic salvage is theoretically possible poststroke [58]. Protein ubiquitination and the Rac signaling may contribute to apoptotic autophagy and inflammation poststroke [59, 60]. In the KEGG analysis, the host genes of circRNAs were also enriched in the cAMP, CREB, MAPK, PI3K-Akt, and HIF signaling pathways, which are associated with pathophysiological mechanisms, such as oxidative stress injury, mitochondrial injury, inflammation, apoptosis, and neuronal regeneration after stroke. The signaling pathways and cell biological processes identified are similar to those reported in previous studies on stroke [47–49]. We found that DECs in the early stages of cerebral ischemia/reperfusion could regulate host gene expression in response to secondary injuries of the poststroke cortex, providing a novel direction for studying the mechanisms of secondary injuries.

circRNAs act as “miRNA sponges” and exert regulatory effects on numerous downstream target genes [25, 32, 33]. In this study, a novel ceRNA regulatory network, harboring 15 circRNAs, 14 miRNAs, and 16 mRNAs, was finally constructed by referring to the expression characteristics of DECs and the experimental miRNA-mRNA interaction data that has been published in the field of nerve damage and repair. This regulatory network is potentially involved in a series of biological events, such as anticoagulant responses, oxidative stress, responses, vasodilation, nerve regeneration, poststroke inflammatory responses, blood-brain barrier damage responses, cerebral edema, cerebral hypoxia, apoptosis, and axonal growth.

The most important stroke-induced factors are cerebral ischemia and hypoxia. In particular, mitochondrial damage caused by hypoxia can dramatically increase the ROS level, which triggers oxidative stress and further exacerbates oxidative stress cascade-induced neurological damage. HIF-1a appears to be deeply involved in the regulation of hypoxic metabolism, and the upregulation of HIF-1a under hypoxic conditions activates miR-182 expression and inhibits the activation of oxygen-dependent HIF-1a degradative enzymes, PHD2 and FIH, resulting in a positive feedback loop that promotes the irreversible activation of HIF-1a [61]. We speculated that circ_Dlgap3_1 and circ_Smad4_4 may specifically adsorb miR-182 and that their significant downregulation led to the enhanced release of miR-182 and the activation of HIF-1a. Moreover, the upregulation of circ_Camta1_9 by 2.11-fold under hypoxic conditions led to the increased absorption of miR-18a-3p, thereby promoting the expression of its target gene *HIF-1a* [62]. Intense oxidative stress stimulates the ROS scavenging mechanisms, and the marked downregulation of circ_Gucy1a2_7 and circ_Ryr2_36 by more than 4-fold after stroke may lead to the release of adsorbed miR-7a-5p and the consequent downregulation of Keap1 at the posttranscriptional level. The decrease in Keap1 expression promotes the entry of Nrf-2 into nuclei and activates the Nrf-2 pathway, which in turn scavenges ROS in the brain to protect brain tissue from oxidative stress-induced damage [63–66].

Neuroinflammatory responses play dual roles in ischemic stroke. A severe inflammatory response can trigger the “waterfall effect” of inflammatory factors, resulting in further cell damage, apoptosis, blood-brain barrier damage, and cerebral edema, whereas a well-timed and moderate inflammatory response exerts a protective effect against stroke. TLR-4, an important regulator of innate immunity, activates the NF-*κ*b pathway via the downstream MyD88 pathway to trigger a poststroke inflammatory storm [67, 68]. Here, we showed that upregulated circ_Camta1_9 had multiple miRNA binding sites that could affect several inflammation-related genes, such as *TLR-4*, *PD-L1*, *AQP-4*, and *Nurr1*. circ_Camta1_9 and circ_Dtnb_10 may act in concert as molecular sponges for miR-140-5p, downregulating its expression and promoting poststroke TLR-4 levels, thereby exacerbating inflammation [67]. Moreover, circ_Camta1_9 may upregulate PD-L1 expression through the adsorption of miR-34a-5p [69]. Interactions of PD-1 with PD-L1 inhibit Treg-mediated neutrophil-derived MMP-9 release after stroke. This inhibition reduces the postischemic breakdown of the blood-brain barrier, leukocyte infiltration, and brain damage [70]. Nurr1 is an orphan nuclear receptor widely distributed in brain tissue that serves as a potent inflammatory regulator in nonneuronal cells, such as microglia and macrophages [71]. In neuroinflammatory processes, the Nurr1-CoREST axis may play an important role in terminating the inflammatory response by clearing p65 from the target promoter [71]. Data from the present study suggested that the upregulation of circ_Camta1_9 and circ_AABR07049790.1_16 inhibited poststroke inflammation through the adsorption of miR-145-5p and the resulting upregulation of Nurr1 [72].

We identified several circRNA host genes enriched in the PI3K-Akt pathway, suggesting that poststroke dysregulated circRNAs have the potential to act on the PI3K-Akt signaling pathways. In bioinformatics analyses, multiple circRNAs affecting the PI3K-Akt pathway were identified. circ_Gucy1a2_7, circ _Snap91_4, circ _Nav2_4, and circ _Camta1_9 were predicted to regulate PTEN through the adsorption of miR-320-3p targets [73]. PTEN suppresses axon and neurite regeneration by inhibiting the activation of the PI3K-Akt pathway [74]. Moreover, miR-320-3p can also target IGF-1 [75]. IGF-1 modulates brain plasticity by affecting neurite growth, synapse formation, neuronal excitability, and neurotransmitter release [76]. The circRNAs originating from the *DGKB* gene (circ_Dgkb_13, circ_Dgkb_14, and circ_Dgkb_10) collectively target SOX9 via miR-124-3p, thus affecting axonal budding after stroke [77].

Apoptosis is the major pathophysiological mode of cell death after stroke, especially in the penumbra immediately adjacent to the ischemic core [11]. In our study, apoptosis-related signaling pathways were simultaneously enriched in GO and KEGG analyses. Bcl-2 is an antiapoptotic protein that inhibits the release of cytochrome C from mitochondria during the apoptotic process by inhibiting BAX and BAK. The released cytochrome C binds to apoptosis protease-activating factor-1 and pre-caspase-9 to form apoptosomes, initiating downstream apoptotic processes [11]. Bcl-2 can be modulated by miR-148a-3p and miR-181a-5p, respectively [78–80]. Our circRNA_miRNA target prediction results showed that circ_Tasp1_7 and circ_Herc3_2 could effectively bind miR-148a-3p and miR-181a-5p, respectively. The above data indicates that circ_Tasp1_7 and circ_Herc3_2 play regulatory roles in apoptosis by entering the BCl-2 signaling pathway. We additionally predicted that the upregulation of circ_Chd2_24 could upregulate apoptosis protease-activating factor-1 by adsorbing miR-23a-3p. Although the specific mechanism has yet to be established, miR-124 also upregulated the antiapoptosis proteins Bcl-2 and Bcl-xl after stroke and exerted antiapoptotic effects [81]. In addition, a circ _Nav2_4/miR-129-5p/HMGB1 signaling pathway was predicted in this study on the basis of their interaction expression patterns and the findings that HMGB1 being a direct target of miR-129-5p [82, 83]. HMGB1 is an important chromosomal protein released outside the nucleus that binds to the receptor forming advanced glycation end products and influencing the inflammatory process after stroke. Finally, with regard to the relationship between neurobehavioral scores and circRNA expression, we conducted a clinical study to analyze the relationship between the expression of key circRNAs and stroke disease severity and prognosis. The results of the clinical trial will be published when they are completed.

Ischemic stroke is a complex multifactorial disease in which the cascade response to ischemia is not a single linear process but often involves parallel or interacting with other mechanisms and events [3, 4, 17]. A series of major events, such as hypoxia, oxidative stress, mitochondrial injury, inflammation, necrosis, and apoptosis, are not only mutually causative, but also exert opposite biological effects of neuroprotection or injury, which ultimately affect the prognosis and outcome of the disease, depending on the combined actions of various factors [21, 84]. Our experiments revealed significant differences in the expression patterns of circRNAs in ischemic stroke. These circRNAs were involved in various mechanisms and events in the ischemic cascade through both the regulations of host genes at the transcriptional level, which affects the poststroke-related signaling pathways involved in major pathological mechanisms, and the ceRNA mechanisms, which regulates downstream target genes at the posttranscriptional level. In view of the dynamic complexity of stroke development, exploring complex network mechanisms involving multiple molecules and pathways, as well as various regulatory levels and factors, may provide a broader perspective for the comprehensive understanding of this disease.

## 5. Conclusions

Our findings collectively demonstrated alterations in the expression patterns of numerous circRNAs in the cortexes of poststroke rats. For the first time, the potential roles of circRNA-miRNA-mRNA and downstream regulatory network pathways involved in oxidative stress, inflammatory response, apoptosis, and regeneration in stroke development have been highlighted. A comprehensive investigation of the changes and functions of circRNAs following ischemic stroke should aid in elucidating the molecular mechanisms underlying stroke and allow the development of effective therapeutic options.

## Figures and Tables

**Table 1:** List of divergent primers designed for RT-PCR validation of circRNAs.

circRNAs	Primer sequence (5′-3′)	Annealing temperature (°C)	Size (bp)
circ-AABR	Forward: TGGGAAGAAAGGGGTGAC	60	265
	Reverse: GTAAAGGGTACTAGAGCC		
circ-Nf1x	Forward: TGTGCGTCCAGCCACATC	60	310
	Reverse: AACTCGGGCCGGATGTCT		
circ-Homer1	Forward: GCAAAGGAGAAGTCGCAG	65	350
	Reverse: TGCCTTTGAGCCGTCTAG		
circ-Chsy1	Forward: TGTACACCACCCACGAGGAT	58	300
	Reverse: CGATCTCCCTTGATGTAGAC		
circ-Snap	Forward: ATGTGCATCCAAATGAGCTA	60	178
	Reverse: TCAAAGAGGGTATCCGCCAT		
circ-Chd	Forward: AAATCGGAGCAGACAAGAACC	55	233
	Reverse: GGCTCTCAGAAGATTCCGAAC		
circ-Wdr-F	Forward: CAGTTTCTCACCCACGGAT	65	198
	Reverse: GCCGCTTATAGAACAACTGTCG		
circ-int3193	Forward: CCCTACAAGAAGCACCATCGT	58	360
	Reverse: AGGCAGTGGGTGTTGTGACT		
circ-Adam	Forward: GAGTACGTGTGGATCACAGG	60	302
	Reverse: GGAGGTTTGTCGAGGACTTC		

## Data Availability

The datasets generated for this study can be found in the Short Read Archive (SRA) of the National Center for Biotechnology Information (NCBI) under the bioproject number PRJNA690203 (https://www.ncbi.nlm.nih.gov/bioproject/PRJNA690203/).

## Supplementary Materials

Supplementary Materials The following are available online. Figure S1: distribution of circRNA reads. Figure S2: tissue enrichment of select genes. Table S1: neurobehavioral scores and performance of the selected rats in the MCAO group and sham group. Table S2: summary of the mapping data from the cortex tissue. Table S3: circRNA_known. Table S4: circRNA_newname. Table S5: DE-circRNAs. Table S6: Biological_Process_enrich. Table S7: Molecular_Function_enrich. Table S8: Cellular_Component_enrich. Table S9: KEGG_pathway_enrich. Table S10: cyto-scape_circRNA-miRNA-mRNA. Table S11: expression of select mRNAs.
