# Supplementary material for: Deep Sequencing of the Rat MCAO Cortexes Reveals Crucial circRNAs Involved in Early Stroke Events and Their Regulatory Networks
Source: Neural Plast. 2021 Nov 24;2021:9942537. doi: 10.1155/2021/9942537 (PMC8635952; doi:10.1155/2021/9942537)
Supplement: Supplementary Materials — The following are available online. Figure S1: distribution of circRNA reads. Figure S2: tissue enrichment of select genes. Table S1: neurobehavioral scores and performance of the selected rats in the MCAO group and sham group. Table S2: summary of the mapping data from the cortex tissue. Table S3: circRNA_known. Table S4: circRNA_newname. Table S5: DE-circRNAs. Table S6: Biological_Process_enrich. Table S7: Molecular_Function_enrich. Table S8: Cellular_Component_enrich. Table S9: KEGG_pathway_enrich. Table S10: cyto-scape_circRNA-miRNA-mRNA. Table S11: expression of select mRNAs. [file 9942537.f1.zip › Table S1 S2.docx]

Table S1 Neurobehavioral scores and performance of the selected rats in the MCAO group and Sham group

| RAT | Longa score | EBST score |
| --- | --- | --- |
| M01 | 1 | 0.90 |
| M02 | 2 | 0.93 |
| M03 | 1 | 0.97 |
| M04 | 2 | 0.97 |
| M05 | 2 | 0.93 |
| S01 | 0 | 0.67 |
| S02 | 0 | 0.4 |
| S03 | 0 | 0.63 |
| S04 | 0 | 0.4 |
| S05 | 0 | 0.57 |

Table S2：Summary of the mapping data from the cortex tissue

| **Items** | **Total Reads** | **Mapped Reads** | **Mapping Rate** | **Uniq Map Reads** | **Uniq Map Rate** | **Multiple Map Reads** | **Multiple Map Rate** |
| --- | --- | --- | --- | --- | --- | --- | --- |
| M01 | 42,935,722 | 42,352,046 | 98.64% | 16,012,545 | 37.29% | 26,339,501 | 61.35% |
| M02 | 43,980,838 | 43,788,910 | 99.56% | 23,498,813 | 53.43% | 20,290,097 | 46.13% |
| M03 | 43,363,406 | 43,197,304 | 99.62% | 21,056,740 | 48.56% | 22,140,564 | 51.06% |
| M04 | 39,943,234 | 39,828,624 | 99.71% | 21,604,896 | 54.09% | 18,223,728 | 45.62% |
| M05 | 42,959,340 | 42,853,708 | 99.75% | 21,881,544 | 50.94% | 20,972,164 | 48.82% |
| F01 | 42,052,314 | 41,776,778 | 99.34% | 22,733,203 | 54.06% | 19,043,575 | 45.29% |
| F02 | 49,977,210 | 49,830,802 | 99.71% | 25,135,377 | 50.29% | 24,695,425 | 49.41% |
| F03 | 42,618,072 | 42,483,016 | 99.68% | 23,476,168 | 55.09% | 19,006,848 | 44.60% |
| F04 | 67,364,602 | 67,106,492 | 99.62% | 34,478,928 | 51.18% | 32,627,564 | 48.43% |
| F05 | 59,169,082 | 58,871,488 | 99.5% | 30,718,688 | 51.92% | 28,152,800 | 47.58% |
