# Supplementary figures and images for: Deep Sequencing of the Rat MCAO Cortexes Reveals Crucial circRNAs Involved in Early Stroke Events and Their Regulatory Networks
Source: Neural Plast. 2021 Nov 24;2021:9942537. doi: 10.1155/2021/9942537 (PMC8635952; doi:10.1155/2021/9942537)

**A**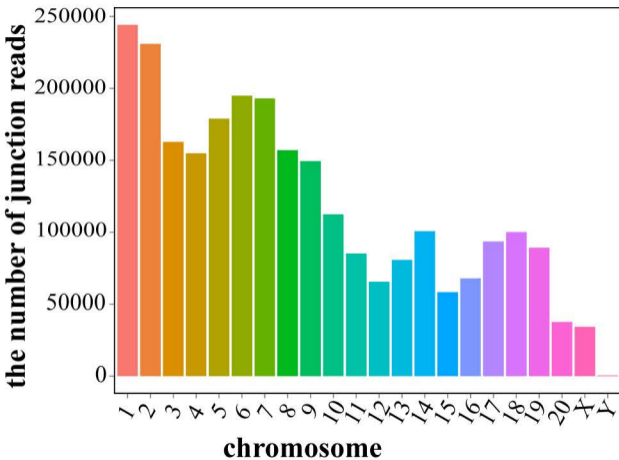**B****CircRNA Length Distribution of All\_circRNA**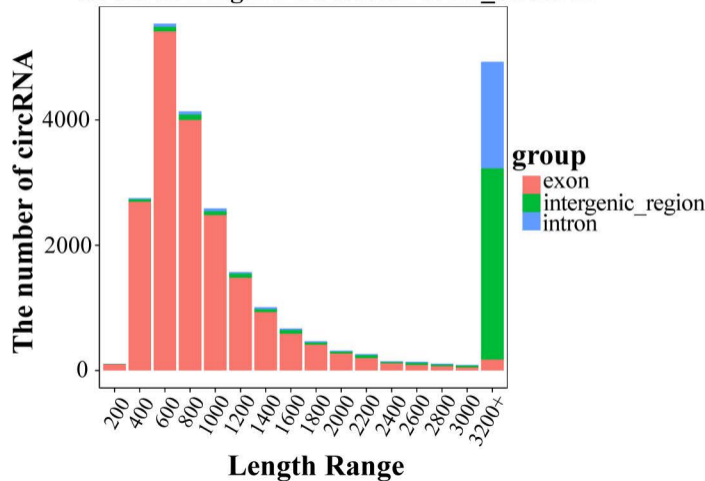

Supplement: Supplementary Materials — The following are available online. Figure S1: distribution of circRNA reads. Figure S2: tissue enrichment of select genes. Table S1: neurobehavioral scores and performance of the selected rats in the MCAO group and sham group. Table S2: summary of the mapping data from the cortex tissue. Table S3: circRNA_known. Table S4: circRNA_newname. Table S5: DE-circRNAs. Table S6: Biological_Process_enrich. Table S7: Molecular_Function_enrich. Table S8: Cellular_Component_enrich. Table S9: KEGG_pathway_enrich. Table S10: cyto-scape_circRNA-miRNA-mRNA. Table S11: expression of select mRNAs. [file 9942537.f1.zip › Fig S1 distribution of circRNA reads.pdf]

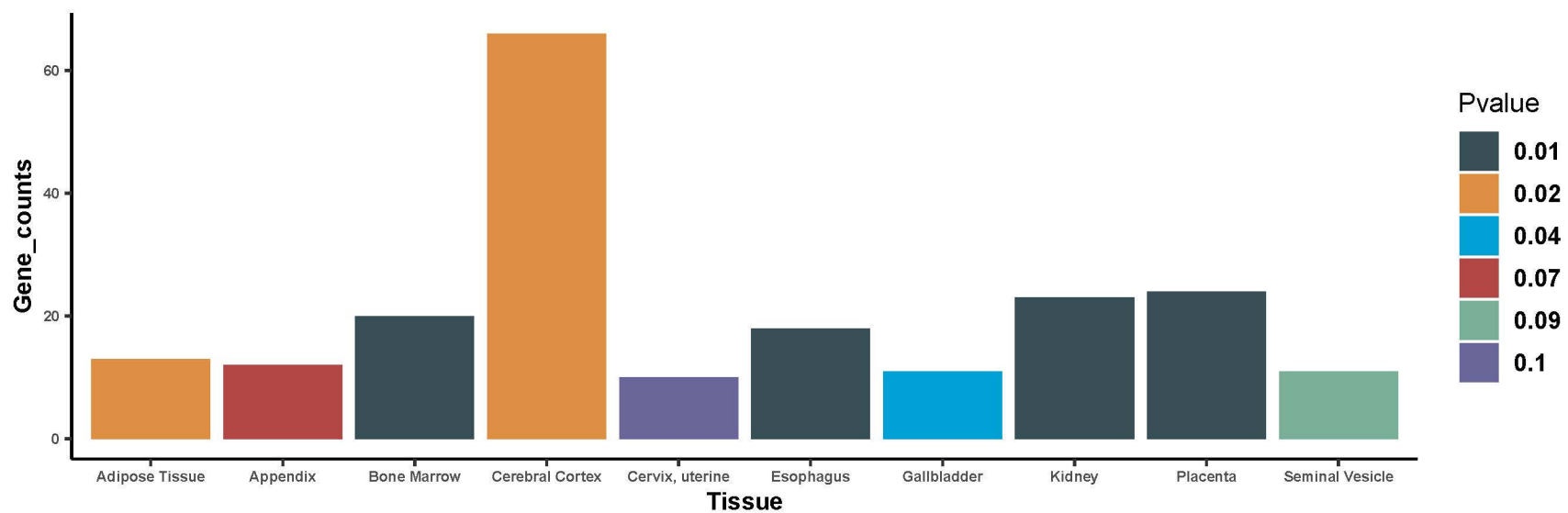

Supplement: Supplementary Materials — The following are available online. Figure S1: distribution of circRNA reads. Figure S2: tissue enrichment of select genes. Table S1: neurobehavioral scores and performance of the selected rats in the MCAO group and sham group. Table S2: summary of the mapping data from the cortex tissue. Table S3: circRNA_known. Table S4: circRNA_newname. Table S5: DE-circRNAs. Table S6: Biological_Process_enrich. Table S7: Molecular_Function_enrich. Table S8: Cellular_Component_enrich. Table S9: KEGG_pathway_enrich. Table S10: cyto-scape_circRNA-miRNA-mRNA. Table S11: expression of select mRNAs. [file 9942537.f1.zip › Figure S2 Tissue enrichment of select genes.pdf]
